# Recent Advances in Antibacterial Coatings to Combat Orthopedic Implant-Associated Infections

**DOI:** 10.3390/molecules29051172

**Published:** 2024-03-06

**Authors:** Seref Akay, Anan Yaghmur

**Affiliations:** Department of Pharmacy, Faculty of Health and Medical Sciences, University of Copenhagen, 2100 Copenhagen, Denmark

**Keywords:** antibacterial, biofilm, infections, local antimicrobial agent delivery, lyotropic non-lamellar liquid crystalline phases, orthopedic implants, polymeric coatings

## Abstract

Implant-associated infections (IAIs) represent a major health burden due to the complex structural features of biofilms and their inherent tolerance to antimicrobial agents and the immune system. Thus, the viable options to eradicate biofilms embedded on medical implants are surgical operations and long-term and repeated antibiotic courses. Recent years have witnessed a growing interest in the development of robust and reliable strategies for prevention and treatment of IAIs. In particular, it seems promising to develop materials with anti-biofouling and antibacterial properties for combating IAIs on implants. In this contribution, we exclusively focus on recent advances in the development of modified and functionalized implant surfaces for inhibiting bacterial attachment and eventually biofilm formation on orthopedic implants. Further, we highlight recent progress in the development of antibacterial coatings (including self-assembled nanocoatings) for preventing biofilm formation on orthopedic implants. Among the recently introduced approaches for development of efficient and durable antibacterial coatings, we focus on the use of safe and biocompatible materials with excellent antibacterial activities for local delivery of combinatorial antimicrobial agents for preventing and treating IAIs and overcoming antimicrobial resistance.

## 1. Introduction

The rise in life expectancy is associated with an increased demand for healthcare among the elderly with hip osteoarthritis or osteoporotic fractures, as well as an increased demand for joint replacement surgeries and development of safe and biocompatible orthopedic biomaterials for combating implant-associated infections (IAIs) [1,2,3]. Here, it is worth considering that the surgical placement of orthopedic implants inside the human body for offering biological support to injured tissues or organs and restoring biological functioning is challenging due to possible bacterial adhesion and proliferation on the surfaces, eventually leading to infections [4,5,6].

Despite extensive recent endeavors in improving biomedical implants through surface modification strategies and the use of biomaterials with excellent anti-biofouling properties for preventing the recruitment of biofouling microorganisms that initiate irreversible attachments on implant surfaces, IAIs still represent a major public health and socioeconomic burden. This is due to the intrinsic tolerance of biofilms to antimicrobial agents and the immune system, and the increased potential of antibiotic resistance development due to long-term and repeated conventional antibiotic treatments [7,8]. The latter is a major health concern, according to the “A European One Health Action Plan against Antimicrobial Resistance (AMR)” report (2017): antibiotic resistance causes approx. 25,000 deaths per year in the EU, and it is estimated to cause more deaths than cancer by 2050. It is also increasing the economic burden across the EU with an annual healthcare cost of about EUR 1.5 billion per year. In the USA alone, IAIs account for 25% of all hospital-acquired infections and the annual healthcare burden exceeds USD 4 billion [9,10,11,12].

Orthopedic IAIs are severe complications resulting in implant failures, often require secondary implant replacement surgeries, and may lead to amputation or mortality, and represent, therefore, a major public health burden due to extended hospitalization, increased time of rehabilitation, and high healthcare costs [13,14]. It is also challenging to treat IAIs due to the typical metabolic heterogeneity of the biofilm-embedded microbial cells, which are not directly targeted by conventional antibiotic treatments [15]. Further, systemic antibiotic delivery is not an efficient therapeutic option as bacterial infections are capable of developing resistance to most widely used conventional antibiotics [1,16]. The clinical incidences of orthopedic IAIs are 2–5% worldwide, and the annual healthcare and treatment expenses are around USD 20 billion [13,15,17].

As the orthopedic implant market is expected to grow, the risk of associated infections should be minimized by preventing bacteria colonization at implants, and addressing therapeutic challenges related to development of biofilms, which are complex dense matrices of proteins, polysaccharides, and DNA-embedding bacteria [12]. The formation of biofilms at implants significantly impairs the efficacy of antibiotic treatment by increasing the antibiotic resistance of internal biofilm cells by 10–1000 times [18]. This necessitates efforts to design safe and efficacious strategies for targeting implant-associated infections and effectively combating the development of biofilms through implant surface modifications and the use of biomaterials with excellent anti-biofouling properties, and assist, therefore, host tissues around the implants in winning *“the race for the surface”* [19,20]. As an alternative to systemic antibiotic use, the FDA has approved various biomaterial candidates that can be used for targeting IAIs [21].

Extensive research has explored various strategies for preventing bacterial adhesion and biofilm formation on orthopedic implants. These strategies, primarily focusing on surface design, can be classified into two groups: strategies for development of surfaces with either passive bacterial repelling or with active bacteria killing [22]. The former strategy focuses on preventing biofilm formation on the implants, whereas the latter focuses on disrupting bacterial cells and achieving efficient bacterial eradication. For achieving efficient action against both adhered and surrounding bacteria, the active bacteria killing strategy focuses on designing safe coatings for orthopedic implants capable of locally releasing antibacterial agents [12,23]. There is recent rapidly growing interest in this strategy for inhibiting biofilm formation on implants due to an increase of occurrence of superbacteria and resistance to conventional antibacterial agents [23].

Considering the increased formation of antibiotic-resistant bacteria embedded on medical implants (particularly orthopedic implants), and the rise of complications associated with the surgical procedures and the long-term use of these implants, there is a recent growing interest in introducing new, safe, and effective strategies for combating IAIs [24]. In this contribution, we focus on recent advances in the development of surface implant modifications and development of antibacterial coatings. We further highlight promising future directions in engineering and functionalization of orthopedic implants with unique structural features, design of orthopedic personalized implants, and new coatings (including the use of self-assembled nanostructures such as inverse non-lamellar liquid crystals in the design of coatings) for local delivery of combinatorial antimicrobial agents for treating infections, overcoming antibiotic resistance development, and stimulating bone tissue regeneration.

## 2. Biofilm Formation on Orthopedic Implants and Prevention Strategies

Bacteria may invade the surgical site and induce infection. There is, therefore, a high risk of bacterial adhesion and colonization on orthopedic implants, leading eventually to biofilm infections [24,25,26]. Similar to other medical devices and implants, most orthopedic implants generally fail due to the development of IAIs and poor tissue interconnections [27,28]. Such poor host integration between the implants and their surrounding bone tissues may lead to the presence of small void spaces around implants, allowing a suitable environment for bacterial adhesion and colonization [5,7,12,26].

**Figure 1:**
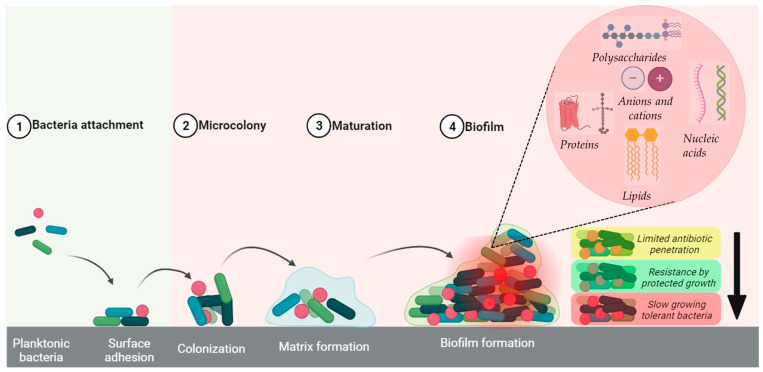
Bacterial colonization and biofilm formation on a medical implant (created with BioRender.com (accessed on 29 February 2024)).

The four-stage biofilm formation process on medical implants is illustrated in Figure 1. The initial preliminary phase is reversible and occurs through the attraction and attachment of microorganisms to the solid surface directly or through interactions with pre-adsorbed proteins [13]. This bacterial adhesion stage paves the way for cell attachment and microbial colonization at the implant, leading eventually to the formation of microcolonies and development of a 3D unique biofilm structure, which is built of hydrated extracellular polymeric matrix (EPM). As these microcolonies grow in density, the produced extracellular biopolymers are effective in encasing bacteria and maintaining the biofilm structure. With bacterial pili and flagella structures, these biopolymers form the EPM [29]. The 3D biofilm structure is comprised of less than 10% of bacterial cells and the remaining part is mainly composed of lipopolysaccharides, polysaccharides, proteins, nucleic acid, and lipids [30,31,32,33]. The EPM provides the biofilm with the following: mechanical stability, enhancement of adherence to the implant surface, and an efficient polymeric network for assessing intercellular communications through signaling routes that exist among the biofilm cells [30]. Biofilms have heterogeneous environments as the superficial layers consist of metabolically active bacteria, whereas the bacteria in the deeper layers become metabolically inactive due to inefficient oxygen and nutrient diffusion into the biofilm matrix [34]. It is worth noting that the metabolically active bacteria can revert to the planktonic forms by detaching from the biofilm matrix, leading to the initiation of new infections in other sites of the body or the implant [30].

The 3D complex biofilm matrix with unique physical and biological properties and inherent morphological features provides the pathogenic bacteria with an exceptional ability to avoid their killing by host defense components and antibiotics. In addition to the poor permeation of biofilms through the EPM network, it is important to consider the plausible inactivation of antibacterial agents (including conventional antibiotics) in the internal acidic and anaerobic biofilm environments [25]. Thus, the adequate antibiotic dosing required for eliminating the encased bacteria can be hampered [32,35]. Alteration in cell metabolism, either through chromosomal mutations or horizontal drug resistance gene transfer, can condense the biofilm and increase its tolerance, which is the capacity of a bacterial colony to endure lethal antibiotic levels in the short term, and resistance to antibiotics [24]. A relatively high bacterial population level in the biofilm matrix enhances interactions among the embedded bacterial cells through biochemical signaling pathways [24]. This close communication is generally associated with a facilitated exchange and horizontal transfer of virulence genes and resistance genes among microorganisms [34,36]. Due to anaerobic stress conditions in the biofilm matrix, some subpopulations of bacteria might develop resistant phenotype states and express biofilm-related antibacterial resistance genes [2]. As a result, conventional antimicrobial agents are unable to completely eradicate the encased biofilm pathogens, as typically applied standard dosing cannot penetrate the biofilm and kill bacteria. Considering the failure and the poor outcomes of traditional systemic antibiotic treatments, further interventions, including surgical implant exchange or removal without replacement, are commonly needed as the only options to eliminate infections. In more violent cases, amputation or lethal consequences may arise due to unrestrained persistent infection around the implant and surrounding tissues [15,22,31,37,38,39].

### 2.1. Biofilm Prevention Strategies

As biofilms are capable of growing on nearly any surface during surgery, it is of paramount importance to introduce efficient strategies to reduce the economic burden and severe consequences of IAIs, including amputation and patient’s pain, and increase survival [25]. These strategies should take into account that the first 5–6 h following surgery are critical as the implants are primarily in good condition for enhancing bacterial attachment and colonization [30,39]. It is important, therefore, to minimize such risk through the use of antimicrobial prophylaxis prior to and post surgery [2]. It is also recommended that the antibiotic treatment should avoid the administration of relatively high doses of antibiotics for an extended period that may result in the development of bacterial resistance [13].

It is worth noting that the implant surface characteristics, including topography, wettability, and surface chemistry, may significantly influence implant–host tissue integration [30]. On implant placement, the host cells and pathogens are generally engaged in “*a race to the surface*” to attach and eventually colonize the implant surfaces. Thus, implant–host tissue integration can stop bacterial adhesion when the host cells occupy the implant surfaces before bacteria. In contrast, biofilm development, leading eventually to IAIs, is expected to occur upon fast and direct contact of the implant surfaces with bacteria. Thus, the affinity of the host cells to the implant surface plays an important role in the long-term success of orthopedic implants [13,30]. However, it is still practically challenging to achieve such success due to the similarity of the adhesion mechanism of the host cells to those of bacteria. In addition to non-specific host cell–implant interactions that might hinder optimal integration, bacterial attachment and colonization on the implant surface or around the surrounding tissues are serious concerns [1].

#### 2.1.1. Implant Surface Modification

Implant surface modification strategies are antibiotic-free and focus on designing implant surfaces with unique properties for achieving unfavorable bacterial attachment and colonization [7,13,27,30,36,40]. Among others, anti-biofouling and contact killing are promising and most investigated viable options for prevention of bacterial adhesion [7]. Here, surface nanotopography can decrease bacterial adherence and bacterial cell attachment by modifying the surfaces and providing them with unique properties such as roughness, surface energy, wettability, and adhesion [41].

##### Anti-Biofouling Surfaces

For blocking the first stage of biofilm development on orthopedic implants and avoiding the use of toxic bactericidal substances, there is a growing interest in the design of implants with anti-adhesive and anti-biofouling properties with relatively very low surface energy. Among others, modifications of the implant surface topography and polymeric immobilization of coatings on implant surfaces are widely investigated in attempts to generate bacteria-repulsive biomimetic surfaces [42,43]. Bacteria are often not entirely eradicated from these surfaces. However, the significant reduction in their numbers on implants hinders bacterial biofilm formation [43,44].

**Figure 2:**
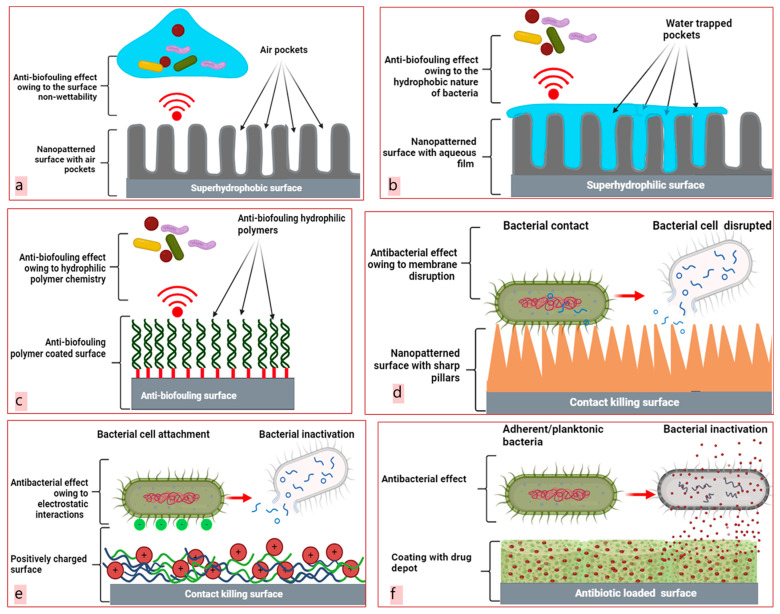
Biofilm prevention strategies. Design of anti-biofouling superhydrophobic surfaces (**a**), anti-biofouling superhydrophilic surfaces (**b**), anti-biofouling polymeric coatings (**c**), nanopatterned contact killing surfaces (**d**), contact-killing polymeric coatings (**e**), and antibiotic coatings (**f**) (created with BioRender.com (accessed on 15 February 2024)).

The design of superhydrophobic surfaces, typically having water contact angles of >150°, is inspired by the surface characteristics of lotus leaves and striders [45,46,47]. Here, the excessive water repellency of such surfaces is attributed to a non-wetting surface chemistry and a high level of roughness, preventing the initial adherence of bacteria that reside in an aqueous environment [7]. As shown in Figure 2a, the anti-biofouling surface characteristics of these implants are attributed to air pockets formed between the surface and the bacterial cell membrane, hindering complete contact [48]. Here, it is worth noting that such surfaces with hierarchical nanostructures can enhance fluid drag reduction and biofouling prevention by retaining trapped air under water [14]. This is attributed to the presence of air pockets significantly reducing the active solid surface area in contact with water, known as the “*Cassie fraction*” [49].

Superhydrophobic surfaces are created by combining rough surface structures with low-surface-energy materials via different approaches [50]. Briefly, rough structures (such as nanopillars, nanochannels, and nanodiscs) are created on hydrophobic materials to obtain superhydrophobicity. Other routes involve the deposition of extremely hydrophobic constituents on substrates to create hierarchically organized surfaces with superhydrophobic layers [43,48,51]. Photolithography, salt etching, sol–gel chemistry, laser ablation and mechanical sand blasting are commonly used to create surfaces with textured arrays and superhydrophobic properties [49,52,53,54]. For example, Manivasagam et al. [55] recently reported on using a simple thermochemical method to modify the characteristics of titanium (Ti) surfaces for preventing biofilm formation on surface with nanotopography, and through a treatment with silane for achieving superhydrophobicity. These silane-modified superhydrophobic surfaces significantly inhibited bacterial colonization (up to 90%), and led to a reduction in the surface adherence levels of *Staphylococcus aureus* (*S. aureus*) and *Escherichia coli* (*E. coli*) as compared to control (unmodified) surfaces.

Another bio-inspired strategy relies on manufacturing superhydrophilic surfaces with water contact angles of <10°. In this strategy, the surface characteristics are modified though chemical alterations, leading to surfaces with unique morphological features by merging regular micro-roughness with hydrophilic substances. Wetting these surfaces leads to water trapping due to surface roughness (known as the *Wenzel wetting state*), and induces therefore the formation of surfaces coated with aqueous layers that effectively restrict bacterial attachment (Figure 2b,c) [56]. As most bacteria interact with surfaces through hydrophobic interactions, increasing the implant surface hydrophilicity is typically associated with an enhanced resistance to bacterial adhesion [14,57]. Polyethylene glycol (PEG) is the most popular anti-biofouling substance as it creates a relatively large exclusion volume on surfaces, leading to the determent of bacterial contamination [58]. In addition, hyaluronic acid and chitosan are attractive for use as hydrophilic modifiers owing to their richness with hydrophilic functional groups, including –OH and ammonium groups. The presence of such groups enhances the surface energy through a significant increase in the hydrophilicity degree of commonly used surface coatings [42,57]. Further studies are needed for gaining further insights into the effects of hydrophilic and hydrophobic modifiers on surface wettability and bacterial adherence [44].

##### Contact-Killing Surfaces

Surface topography is a successful tool for preventing the development of microbial biofilms on implants by killing and physically inactivating attached bacteria [7,14]. In addition to the topographic features at the nanoscale, bacterial cell-surface contact plays an important role in regulating physical damage, which is known in the literature as a contact killing. This biomimetic approach is inspired by the structures of cicada and dragonfly wings, which are decorated with nanospikes that act as defensive coatings against microbial contaminants [59]. In this research area, analogous topographic nanostructures, including nanorods, nanopillars, and nanoedges, are constructed to acquire inherent bacterial-killing properties [42]. The simple mechanism of contact killing by surface topography is presented in Figure 2d. The dimensional properties (such as height, width, and spacing) of these nanostructures have decisive roles in the contact-killing mechanism [41]. Nanotextured surfaces, with thick, blunt nanopillars, promote bacterial adhesion between spikes, causing cell body suspension, stress, and rupture, ultimately leading to cell death in bacteria [25,60]. To generate unique 3D topography-patterned surfaces with nanopillars, many processes such as electrochemical anodization, hydrothermal etching, and 3D direct laser writing are used [42]. However, this mode of action based on the disruption of the cell membrane by surfaces with nanopillars may not be similarly successful against certain cells with relatively thicker or larger cell walls or those having an additional membrane [61]. Recently, biomolecule-based contact-killing techniques have been introduced as more sustainable alternatives to topographic surface nanopatterning. In these studies, the utilization of different biopolymers (including chitosan and cellulose) and various antimicrobial peptides (AMPs) has been reported [7,58,62]. The antibacterial activities of these cationic biomolecules are most likely attributed to their electrostatic interactions with typically negatively charged bacterial membranes, resulting in cell content leakage and eventually microbial death (Figure 2e) [2,14,20,34]. The recent increasing interest in the design of coatings with sustained release properties of AMPs is attributed to their advantages as compared to typically used antibiotics. Small cationic peptides can penetrate or travel across negatively charged bacterial membranes, generating small pores that cause burst of cell contents and bacterial death [63,64]. Therefore, AMPs offer exciting alternatives to traditional antibiotics owing to their rapid and non-specific antibacterial mechanisms, which are also less likely to induce bacterial drug resistance. Further, it is worth considering that AMPs are part of the immune system, act as immunomodulating agents, and may exhibit potential biological activities (such as anticancer, antiviral, and antifungal activities) [65,66].

Contact-killing strategies can kill pathogens by preventing direct bacterial contact with the implant surface. Thus, they minimize the risks of biofilm development and bacterial infections. However, these strategies are not active on planktonic bacteria. As the contacted bacteria are ruptured, bacterial debris and intracellular contents can accumulate on the contact surface, diminishing the bactericidal effect of the implant surface. Such an accumulation barrier may form active functional groups and provide binding sites for adhesion of planktonic bacteria [25].

#### 2.1.2. Release-Based Antibacterial Coatings

The design of antibacterial coatings on orthopedic implants has merged as a promising option for the prevention of IAIs (Table 1) [38]. Here, single or combined antibacterial agents (such as antibiotics and AMPs) can be released in sustained-release manners to maintain their relatively high local concentrations on and around the implants and the surrounding host tissues, prevent rapid depletion of antibacterial activity, lower postsurgical contaminations, and kill adherent and adjacent planktonic bacteria (Figure 2f) [6,25,26,43,49,67].

In addition to polymeric antibacterial coatings, various studies have reported on coatings based on lipids and those developed from nanoparticles [26,68,69,70]. Owing to their potential responsivity to external stimuli (including magnetic field, temperature, and light) and typical strong antibacterial effects, metallic nanoparticles are one of the most utilized nanomaterials for antibacterial coatings, either by direct immobilization or embedding in polymeric layers [71,72]. Most coatings are developed from biopolymers with molecular and functional versatilities, allowing the development of coatings with the desired properties and enabling their functionalization with bioactive moieties or antibiotics [6,7,27]. Among the most used biopolymers for coating orthopedic implants, we mention alginate, collagen, cellulose, gelatin, chitosan, hyaluronic acid, and synthetic polymers such as polycaprolactone (PCL), polyetheretherketon (PEEK), poly-L-lactide (PLLA), poly-D,L-lactide-co-glycolide (PLGA), polyurethane, and poly(vinyl alcohol) (PVA) [73,74,75]. Here, it is worth mentioning that PLLA-based antibiotic-releasing coatings are already clinically used and commercially available for preventing bacteria colonization on orthopedic implants [30,76].

Owing to their simplicity and efficacy, polymeric coatings with sustained antibacterial agent release properties are a popular choice for preventing IAIs on various orthopedic implants [27,77]. Further, these antibacterial coatings can be composed of multifunctional biopolymers, enabling more sustained release of the loaded antibacterial agents, and resulting in improved integration at the implantation sites. In another approach, multifunctional coatings are produced through embedding soft or hard nanoparticles in the polymeric matrices intended for use in the implant coating [6]. For example, Song et al. [78] reported on the development of antibacterial coatings based on chitosan loaded with nanospheres containing vancomycin or moxifloxacin. In another study, Xu et al. [79] reported on the immobilization of minocycline-free and minocycline-encapsulated liposomes on a polystyrene surface through polydopamine conjugation for potential implant coating applications. In the presence of minocycline, these coatings exhibited anti-biofilm activities against *Porphyromonas gingivalis* and *Streptococcus mutans* via a significant decrease (up to 98%) in the bacterial adhesion on the implants. There was also a significant decrease (up to 92%) in the bacterial adhesion, when coating the implants with the control (minocycline-free liposomes) polymeric matrix. This is attributed most likely to the properties of the developed implant coating, leading an increase in the hydrophilicity level of the implant’s surface.

In most investigated polymeric coatings, the loaded bioactive payloads are embedded through either a physical entrapment method or a chemical conjugation (an attachment to coatings via chemical bonding) method. The sustained release properties of these payloads are affected, among others, by the employed coating development method, and the coating’s composition, charge, and structural features [25,80]. For instance, the release mechanism for physically encapsulated components in coatings is generally passive and affected by various factors, including polymer charge and concentration, affinity to the implant surface, crosslinking degree, degradation rate, and swelling behavior of the polymeric matrix, as well as the drug’s physiochemical properties and its concentration [73]. For most polymeric coatings, antibacterial agent release kinetics have been shown to follow first- or second-order kinetics, after a typical initial burst release behavior [6,25]. However, designing a biopolymeric coating on implants can be challenging due to the possible fast release of the initially loaded antibacterial agents, leading to a significant decrease in their antibacterial effects [6,45]. Hence, it is generally problematic to maintain the local concentration of antibacterial agents above the minimum therapeutic concentration for required prolonged periods [25]. Further, variables such as polymer degradation and early release caused by various physiological circumstances may impair coating effectiveness on the implants [6,45].

In recent years, hydrophilic interpenetrating 3D polymeric networks, known also as hydrogels, were reported as suitable materials that can be used in the development of antibacterial coatings [76]. Owing to the adjustability of the intercommunicating networks of hydrogels, they are attractive for loading antibacterial agents with various molecular weights and physiochemical properties and sustaining their release around the implantation site [25,28]. Among the biopolymers used in the formation of hydrogels, biologically relevant materials, including collagen, gelatin, hyaluronic acid, chitosan, and alginate, are widely used owing to biodegradability, safety, and their capabilities of inducing cell adhesion, growth, and differentiation [28,81,82]. Among previous studies, Wu et al. [83] reported on utilizing binary combinations of chitosan and gelatin for producing hydrogel coatings on Ti implants, facilitating bone cell adherence and growth. Prevention of microbial infections on the implants was achieved through loading these coatings with antibacterial silver nanoparticles (AgNPs). In another study, Zarghami et al. [84] reported on the development of composites loaded with vancomycin and melittin from combinations of chitosan and bioactive glass nanoparticles. Their use as coatings was associated with an enhanced osteoblast cell proliferation and an effective elimination of both of methicillin-resistant *S. aureus* (MRSA) and vancomycin-resistant *S. aureus* (VRSA) bacteria. Huang et al. [28] also reported on the use of hydrogels loaded with vancomycin for coating 3D-printed Ti scaffolds. Coating these chitosan–hyaluronic acid hydrogels on Ti scaffolds led to more sustained release of vancomycin under initial conditions, and a significant inhibition of MRSA adhesion and colonization on the implant surfaces. Regarding orthopedic implant coating with hydrogels, it is worth taking into account that their swelling degree and macroscale coating thickness may restrict their attachment and durability on these implant surfaces [85].

In addition to hydrogels, there is a growing interest in the development of coatings with sustained drug release properties from polyelectrolyte multilayers (PEMs), which are nanostructured polymeric layers of opposing charges, or from polymeric nanofibers [43]. In a recent study, Yavari et al. [86] reported on vancomycin-loaded coatings composed of PEMs that were produced from chitosan and gelatin for modified Ti implants. They were efficient in sustaining vancomycin release and eradicating both of planktonic and adhered microorganisms on the implants. In another study, Mathur et al. [87] recently reported on development of coatings from gelatin nanofibers with embedded AgNPs to modify a Ti surface. They exhibited unique antibacterial activities against *E. coli* and *S. aureus*, with 99.99% elimination for a prolonged period of 48 h.

**Table 1:** Examples on developed antibacterial polymeric coatings for orthopedic implants.

Coating Type	Coating Method	Coating Materials	Antibacterial Agent	Outcome	Ref
Release-based	Direct injection	Hyaluronic acid and carboxymethyl chitosan	Vancomycin	Inhibition of free and adherent bacteria on Ti surface through use of hydrogel coatings loaded with vancomycin	[28]
Release-based/contact killing	3D printing	Chitosan and gelatin	Chitosan and nano-silver solution	Hydrogel coatings with embedded AgNPs on Ti surface, having strong antimicrobial activity against *E. coli* and *S. aureus*	[83]
Release-based	Drop-casting	Chitosan, bioactive glass, and melittin	Vancomycin	Coatings loaded with vancomycin or melittin, having strong antimicrobial activity against vancomycin-resistant *S. aureus*. They inhibited biofilm development on the Ti implant surfaces	[84]
Release-based	Layer-by-layer coating	Gelatin and chitosan	Vancomycin	Coatings loaded with vancomycin showed antibacterial activity against planktonic and adherent *S. aureus* on Ti implant surfaces	[86]
Release-based	Air-brush spraying	Poly-D,L-lactide (Resomer^®^)	Vancomycin, Al_2_O_3_ nanowire, and TiO_2_ nanoparticles	Vancomycin-loaded coating, preventing formation of resistant *S. aureus* biofilm on Ti discs	[88]
Stimuli-responsive	Direct covalent linkage	Lecithin, cholesterol and PEGylated DSPE	IR780 and perfluorohexane	Liposome coatings, which were produced through covalent linkage on Ti implants, had a strong antibacterial effectiveness against *E. coli* (99.62%) and *S. aureus* (99.63%)	[89]
Release-based	Layer-by-layer coating	Vaterite and alginate	Vancomycin	Coatings with vancomycin sandwiched between layers of vaterite on Ti surface, having good antimicrobial activity against resistant *S. aureus* up to 7 days	[90]
Release-based	3D printing	Poly- D,L-lactide-co-glycolideand poly(ε-caprolactone)	Vancomycin	Coatings loaded with vancomycin on Ti implants, exhibiting a tunable release above the minimum inhibitory concentration, demonstrating its activity against *S. aureus*	[91]
Release-based	Spray coating	Poly- D,L-lactide-co-glycolide	Gentamicin	Coatings loaded with gentamicin on stainless steel implants effectively inhibited biofilm formation for *S. aureus* and *S. epidermidis*	[92]
Release-based	Layer-by-layer	Poly- D,L-lactide-co-glycolideand gelatin methacryloyl	Cathelicidin-2	Polymeric coatings loaded with Cathelicidin-2 on Ti implants effectively eradicated *E. coli* and *S. aureus* for up to 4 days	[93]
Release-based	Electrospinning	Poly- D,L-lactide-co-glycolideand poly(ε-caprolactone)	Rifampicin and vancomycin	Bi-layer coatings loaded with combinations of antibiotics exhibited sustained antibiotic release against planktonic and adherent *S. aureus* for 6 weeks on Ti implants	[16]
Release-based	Electrospinning	Poly- D,L-lactide-co-glycolideand poly(ε-caprolactone)	Rifampicin, vancomycin linezolid, and daptomycin	Antibiotic-loaded coatings, preventing *S. aureus* infection and biofilm formation on Ti implants	[94]
Superhydrophilic coating	Layer-by-layer	Tannic acid, hydroxyapatite, and PEG		Highly hydrophilic PEG coating with a strong anti-biofilm activity. No biofilm formation by *S. aureus* and *E. coli* on the tested Ti plates	[95]

### 2.2. Techniques for Coating Orthopedic Implants

Despite advancements in fabrication techniques, the use of coatings of orthopedic implants, particularly those produced from polymers, is still challenging. This, among other factors, is attributed to their mechanical non-resistance property and poor durability [43]. Consequently, a fast degradation of the coating combined with non-optimal antibiotic release may lead to the development of antimicrobial resistance, toxicity of the used coating materials, and potential activation of the immune system that may promote inflammatory [96,97]. Thus, there is a growing interest in the development of safe coatings with improved durability and excellent anti-biofilm activities. Further, there is a recent interest in introducing robust strategies and the use of relatively cheap and simple materials in the design of coatings for orthopedic implants [43]. These strategies are mainly focusing on surface chemistry for designing resilient coatings. In this section, we describe recent advances and report on the most used coating techniques, including electrochemical deposition, layer-by-layer coating, and spin coating [6,7,98]. Here, electrospray deposition [99,100], electrospinning [75,101], and electrophoretic deposition [102,103] are promising tools for developing biocompatible and antibacterial coatings on orthopedic implants, including multifunctional coatings.

Another method commonly used for coating orthopedic implants is based on formation of multilayer assemblies on the implants, known as layer-by-layer (Lbl) method. This method is effective for non-covalent implant surface modification. However, covalent bonds may be directly incorporated into LbL multilayers to increase the endurance and stiffness of the produced coatings. Dopamine is commonly used as a covalent linker to provide strong attachment between the implant surface and the LbL multilayers [14,104,105]. Formation of LbL assemblies through electrostatic interactions has been shown to be effective in terms of antibacterial agent loading capacity and durability on implants under various environmental conditions [102]. Owing to their biocompatibility and bioactivity, most cationic and anionic polysaccharides, including chitosan, hyaluronic acid, and alginate, are appropriate materials for use in this method [14]. The charges and functional groups of polysaccharides are used to enhance the electrostatic interactions among the layers of and, these coatings with the implant surfaces. The properties of these materials may also play an important role in modulating the bactericidal activity. Crosslinking between the polymer chains in presence of different functional moieties generally improves the coating’s mechanical properties [14].

The Lbl method is attractive for the design of biocompatible coatings for local delivery of single or combinatorial antimicrobial agents, typically through deposition of inversely charged polyelectrolytes, polymers, or their combinations, on the implants [39]. The antibacterial agent release properties of these coatings can be modulated through alterations in the layer’s type and thickness, variations in number of multilayers, and their biodegradation rates [86]. This method is simple and can be combined with other methods (such as spinning, spraying, and dipping) in the development of coatings [39,86].

Among other coating methods, we briefly mention dip coating and spin coating. The former simple method can be combined with additional steps (such as curing or sintering) for producing coatings on implants with a thickness ranging between 0.02 to 50 µm; whereas the latter method is based on the application of centrifugal forces for producing uniform coatings with a thickness typically ranging between 0.03 and 2 µm on flat surfaces [14,73,106]. It is worth noting that the spin coating is widely used in the development of coatings for biomedical implants. However, its use is associated with different limitations, including the poor adhesion on the implants, generally the poor uniformity on the curved surfaces, and the implant dimensions are limited as they controlled by the spinning device size [3,14,107].

## 3. Current Limitations and Future Directions

Among the most promising strategies for the prevention of orthopedic implant-associated pathogens, we mention contact-killing and anti-biofouling strategies as effective tools for elimination of the initial invasion stage of the orthopedic implant by pathogens [49]. However, their mechanical and physicochemical stabilities can be significantly affected on exposure to the biological environment at the implantation sites, and a lower efficiency may be achieved in vivo as compared to that reported during their in vitro evaluation investigations. Here, particular attention should be given to plausible chemical or hydrodynamic interactions of the implants with biomolecules (mainly proteins) or evolvement of inactivated bacteria debris, leading to a significant decrease in the antibacterial efficiency of the coatings [108]. It is also worth noting that both strategies do not have any effect on the planktonic bacteria, and therefore a complete elimination of the infection risks is not expected. It is possible to overcome such limitations through combinations of antibacterial coating strategies. For example, there are reports on combining anti-biofouling (or contact-killing strategies) with the below-mentioned strategy, focusing on the development of coatings with sustained drug release properties [49].

For inhibiting the infections from adherent and planktonic bacteria, there is also a recent interest in design of antibacterial coatings with sustained drug release properties. Among others, hydrogels have the potential in implant coating development for preventing IAIs, owing to their possible functionalization and long-term performance [20,109,110]. In general, coatings with sustained drug release properties are attractive for avoiding possible bacterial resistance and minimizing systemic effects at the implantation site, through sustained release of single or combinatory antibacterial agents to achieve the required local therapeutic concentrations [111]. Despite their attractiveness in sustaining the release of antibacterial agents (including conventional antibiotics) as compared to contact killing and anti-biofouling strategies, their efficacy and duration are limited and mainly depend on the physiochemical properties of their loaded antibacterial agents, their initial concentration in coatings, and their release rates [20,49,109,110]. Further, their use may be associated with structural instability and fast degradation in the presence of enzymes [110].

**Figure 3:**
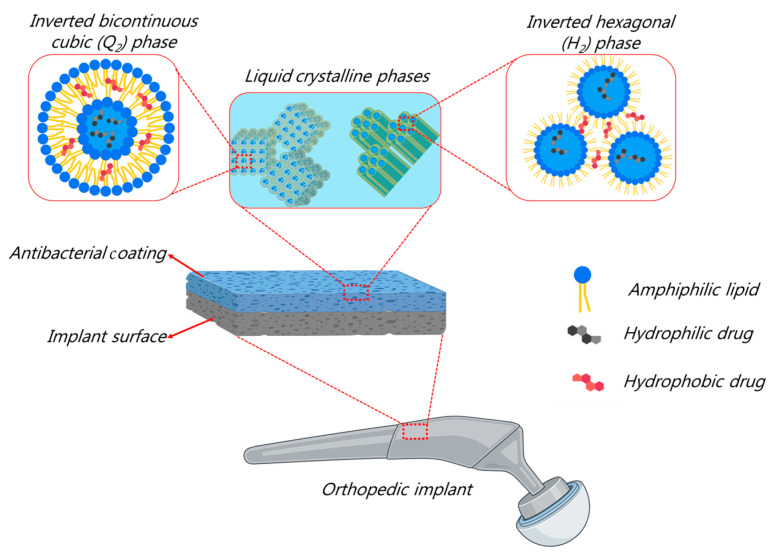
**Non**-lamellar liquid crystalline phases (Q_2_ and H_2_ nanostructures) for designing next-generation safe and efficient antibacterial coatings (created with BioRender.com (accessed on 15 February 2024)).

In the development of safe, multifunctional, and efficient coatings for the prevention of bacterial biofilm infections on orthopedic implants, recent advances focus on exploring biomimetic approaches through the use of naturally occurring compounds or structures in the design of coatings or in implant surface modifications by chemical or topographical processes [41]. Among others, lamellar and non-lamellar liquid crystalline phases, particularly inverse bicontinuous cubic (Q_2_) and hexagonal (H_2_) phases, may find application in next-generation antibacterial coatings, as depicted in Figure 3. This is attributed to their unique nanostructural versatility, the biocompatibility of their major lipid constituents (such as monounsaturated monoglycerides, diunsaturated monoglycerides, and omega-3 fatty acid monoglycerides), bioadhesive properties, and capability of loading and sustaining the release of amphiphilic, hydrophobic, and hydrophilic drugs [112,113,114,115,116,117,118,119,120,121,122,123]. Further, it is worth exploring the possible functionalization of hydrogels or polymeric matrices by embedding the corresponding nanoparticles of the inverse non-lamellar lyotropic liquid crystalline phases (particularly cubosomes and hexosomes), which are recently popular nano-self-assemblies in the development of nanocarriers for drug delivery applications [124,125,126,127,128,129,130,131,132,133,134,135,136,137,138,139,140,141,142], or through their immobilization for designing cubosome or hexosome coatings by employing chemical surface activation methods. The latter strategy is typically applied for immobilization of lamellar liquid crystalline nanoparticles (liposomes) and various soft and hard nanoparticles (such as silver nanoparticles) [49,70,72,105]. The design of such antibacterial coatings through the use of non-lamellar phases is rarely investigated [143]. Among the few published reports, Zabara et al. [65] reported on the formation and characterization of liquid crystalline coatings for silicon wafers, which are loaded with the antimicrobial peptide LL-37 and based on monoolein. They had antibacterial activity against Gram-negative and Gram-positive bacteria.

In addition to the recent progress in the development of antibacterial coatings, it is worth highlighting recent advances in the surface design of modified orthopedic implants with antibacterial properties. There are different future opportunities for implant surface modifications, owing to the recent enormous advances in development of nanofabrication tools (including 3D printing/patterning) and their applications. For instance, future investigations may focus on designing next-generation implants through the creation of antibacterial surface nanostructures with controlled size characteristics and geometrical features. Here, it is worth mentioning that the design of such nanopatterned antibacterial orthopedic implants, having anti-biofouling properties and enhancing superhydrophobic implant–bacteria interactions, is promising for effectively preventing biofilm formation on the implants through a direct contact with bacteria, leading to a significant increase in its oxidative stress [45,144,145]. Those nano-protrusions cause bacterial cells to rupture without inducing any resistance, and without significant harm to mammalian cells [146]. Moreover, nanopatterned antimicrobial surfaces are effective against both Gram-positive and Gram-negative bacteria without involving any antibacterial agents [145,146,147]. Such nano-patterned surfaces can be combined with soft materials to obtain more effective and long-lasting antibacterial coatings, and they may be used as innovative nanocatalytic therapies. These therapies are based on supporting these coatings with catalytic components, leading to a significant increase in the local oxidative stress on bacteria [148,149].

It is worth also highlighting, recent advances focusing on utilization of nanofabrication techniques and additive manufacturing for designing personalized orthopedic implants [27]. Here, 3D printing tools are attractive for manufacturing personalized and functionalized orthopedic implants (including titanium systems) with unique structural and morphological features. For instance, these tools can be used to produce multifunctional coatings on nanopatterned implants with strong mechanical properties for precisely loading antibacterial agents (in single or combinatory forms) and sustaining their release. The interest in designing such personalized implants relies on the need to produce safe systems, offering high antibacterial efficacy, improving patient convenience, and providing flexibility according to the patient’s needs. The latter can be achieved by adjusting the implant’s structural and morphological features, mechanical properties, and improving the loading efficacy of antibacterial agents [92,150,151].

In summary, there is a growing interest in both of surface modification and design of safe coatings for orthopedic implants for overcoming major complications and limitations through minimizing size toxic effects, improving stability and durability of coatings, ensuring long-term efficacy, preventing biofilm formation, and focusing on a successful and biocompatible implant integration within surrounding the tissues. In addition to implant surface modification studies, it is promising to develop safe and efficient antibacterial coatings for orthopedic implants. Considering the multidisciplinary nature of this research area, the future investigations will continue focusing on the integration of scientists with different backgrounds (including engineers, chemists, and biologists) for introducing, among others, surface nano-engineered implants with inherent structural and morphological features and safe coatings with enhanced durability on the orthopedic implants. Further collaborations through strategic and joint academy–industry initiatives will be required for designing personalized 2D and 3D orthopedic implants and overcoming current limitations.

## Data Availability

Not applicable.
